# Identification of a MicroRNA Signature for the Diagnosis of Fibromyalgia

**DOI:** 10.1371/journal.pone.0121903

**Published:** 2015-03-24

**Authors:** Germán Cerdá-Olmedo, Armando Vicente Mena-Durán, Vicente Monsalve, Elisa Oltra

**Affiliations:** 1 Facultad de Medicina, Universidad Católica de Valencia “San Vicente Mártir”, Valencia, Spain; 2 Cátedra Umivale en innovación e investigación en patologías del trabajo, Valencia, Spain; 3 Instituto Valenciano de Patología (IVP) de la Universidad Católica de Valencia “San Vicente Mártir”, Centro de Investigación Príncipe Felipe (CIPF), Valencia, Spain; National University of Singapore, SINGAPORE

## Abstract

**Background:**

Diagnosis of fibromyalgia (FM), a chronic musculoskeletal pain syndrome characterized by generalized body pain, hyperalgesia and other functional and emotional comorbidities, is a challenging process hindered by symptom heterogeneity and clinical overlap with other disorders. No objective diagnostic method exists at present. The aim of this study was to identify changes in miRNA expression profiles (miRNome) of these patients for the development of a quantitative diagnostic method of FM. In addition, knowledge of FM patient miRNomes should lead to a deeper understanding of the etiology and/or symptom severity of this complex disease.

**Methods:**

Genome-wide expression profiling of miRNAs was assessed in Peripheral Blood Mononuclear Cells (PBMCs) of FM patients (N=11) and population-age-matched controls (N=10) using human v16-miRbase 3D-Gene microarrays (Toray Industries, Japan). Selected miRNAs from the screen were further validated by RT-qPCR. Participating patients were long term sufferers (over 10 years) diagnosed by more than one specialist under 1990 American College of Rheumatology criteria.

**Results:**

Microarray analysis of FM patient PBMCs evidenced a marked downregulation of hsa-miR223-3p, hsa-miR451a, hsa-miR338-3p, hsa-miR143-3p, hsa-miR145-5p and hsa-miR-21-5p (4-fold or more). All but the mildest inhibited miRNA, hsa-miR-21-5p, were validated by RT-qPCR. Globally, 20% of the miRNAs analyzed (233/1212) showed downregulation of at least 2-fold in patients. This might indicate a general de-regulation of the miRNA synthetic pathway in FM. No significant correlations between miRNA inhibition and FM cardinal symptoms could be identified. However, the patient with the lowest score for mental fatigue coincided with the mildest inhibition in four of the five miRNAs associated with the FM-group.

**Conclusions:**

We propose a signature of five strikingly downregulated miRNAs (hsa-miR223-3p, hsa-miR451a, hsa-miR338-3p, hsa-miR143-3p and hsa-miR145-5p) to be used as biomarkers of FM. Validation in larger study groups is required before the results can be transferred to the clinic.

## Introduction

Fibromyalgia (FM) (ICD-10 diagnosis code M79.7) is defined as a chronic disorder of unknown etiology characterized by low pain threshold, stiffness and tenderness in the muscles of neck, shoulders, back, hips, arms, and legs, usually accompanied by headaches, fatigue, sleep disturbances, memory loss and painful menstruation [1,2]. Recent epidemiological studies estimate its global prevalence at 2–8% with a female/male ratio predominance [3,4]. The devastating situation of sufferers and the high socioeconomic expense that this disease supposes is worrisome.

Currently, the FM diagnosis is made solely on clinical grounds [1], as no validated biological markers associated with the disease have been identified. Alterations in cytokine profiling, decreased response of peripheral blood mononuclear cells (PBMCs) to mitogens and presence of autoantibodies have been reported in these patients [5–7]. Since many of the symptoms characterizing FM such as fatigue and flu-like symptoms resemble those of infectious diseases many authors have investigated a possible viral etiology of the disease [8–13]. However, up to date no clear correlation between FM and viral infection has been established [8,9,14–17]. Identification of markers consistently associated with this pathology will enable clinicians to effectively diagnose FM, follow the progress of the disease, monitor the effects of therapeutic approaches and probably develop preventive programs.

MicroRNAs or miRNAs have been identified as important modulators of gene expression in tissue-specific physiologic pathways, in response to environmental cues and in disease [18–20]. In their mature form, they are short RNA molecules (20–22 nts-long) capable to inhibit translation by inducing degradation of their target coding transcripts [18]. However, miRNA-mediated upregulation of translation has also been reported [21]. In either scenario, a single miRNA can regulate the expression of several genes, and conversely, a transcript can be regulated by more than one miRNA. Thus, changes in miRNA relative abundance may provide signatures of complex gene expression network deregulations commonly found in chronic diseases. This together with their high stability both inside the cell and in body fluids, makes these small nucleic acids attractive biomarker candidates for complex diseases of unknown etiology such as FM.

Two recent studies have reported disease-specific patterns of miRNAs in FM patients. In one of them the authors evaluated miRNA profiles of cerebrospinal fluid [22]. Blood is among one of the easiest biofluids to obtain and therefore suitable to assay in the clinic. This is probably the reason these same authors have more recently evaluated circulating miRNA profiles in serum of FM patients. The qPCR assay they used, however, limited the analysis to only 374 miRNA sequences [23] which represent only 14% of the 2661 human miRNA sequences available in the last 21 version release of miRbase (http://www.mirbase.org/) [24,25].

The aim of this study was to identify miRNA profiles through genome-wide broad scope microarray technology which could be used as a quantitative diagnostic method of FM performed under minimally invasive procedures. These disease-associated miRNAs should serve as general indicators of the affected molecular pathways in FM. In addition, evaluation of the cells selected in the study, PBMCs, should lead to a deeper understanding of the etiology and/or symptomatology of this complex disease provided the immune system defects and/or recurrent infection hypotheses are confirmed.

## Methods

This study was approved by the Hospital de La Plana (Villarreal, Spain) Ethics Committee. A single sample of whole blood was obtained from each individual after signing an informed consent form.

### Patients and healthy controls

Eleven FM patients and ten healthy participants individually matched by age (range +/- 5 yrs.) were evaluated in this study. All eleven patients came from a single Institution, a local FM patient association, Asociación Valenciana de Afectados de Fibromialgia (AVAFI), in Valencia, Spain and met the criteria for FM diagnosis according to the American College of Rheumatology 1990 criteria [1]. Healthy matched controls were regular blood donors from the Valencian Community Blood Bank. Each participant underwent a thorough clinical interview to assess clinical criteria and severity of FM using standardized FM Impact Questionnaire (FIQ) case report forms [26,27]. For fatigue assessment the Multi-fatigue inventory MFI was used [28]. Those with a score ≥ well population medians on the general fatigue or reduced activity scales of the MFI were considered to meet fatigue criteria of the 1994 international case definition. Patient data were entered into a computer database (Microsoft Access 2003, Redmond, WA: Microsoft Corp.).

### Isolation of PBMCs

Blood samples collected and kept at RT in 170 IU of lithium heparin vacutainers (Becton Dickinson BD 365725) were processed within 2 h by dilution at 1:1 (v/v) ratio in phosphate-buffered saline solution (PBS), layering on top of 1 vol of Ficoll-Paque Premium (GE Healthcare) and separation by density centrifugation at 20°C at 500 g for 30 min (brakes off). The PBMC layer was isolated and washed with PBS. The isolated PBMC pellets were resuspended in 1 vol of red blood cell lysis buffer (155 mM NH4Cl, 10 mM NaHCO3, pH 7.4, 0.1 mM EDTA), kept on ice for 5 min, and centrifuged (20°C, 500 g, 10 min), as previously described [29]. The washed pellets were resuspended in freezing medium (90% FBS, 10%DMSO) adjusting their concentration to 10^7^mononuclear cells/ml, aliquoted and frozen at -150°C until use.

### RNA isolation from PBMCs

Total RNA was isolated from frozen PBMCs aliquots rapidly thawed at 37°C and washed in 5 vol PBS, by using the mirVana miRNA isolation kit (LifeTechnologies, cat. AM1560), following manufacturer´s recommendations. RNA concentration and purity were determined by their absorbance at 230, 260 and 280 nm in a Nanodrop 2000 instrument (Thermo Scientific). Integrity of the RNA samples was evaluated by electropherogram analysis in a 2100 Bioanalyzer with Agilent 2100 Expert Software (Agilent Technologies) using total and small RNA kit cartridges at the Unidad de Genómica del Servicio Central de Soporte a la Investigación Experimental (SCSIE) de la Universidad de Valencia (Valencia, Spain). Only samples with a RIN (RNA Integrity Number) over 7.0 or with an intact 28S/18S gel profile were further used. Results of the 21 analyzed samples are shown in S1 Table.

### Microarray Analysis

Extracted total RNA was labeled with Hy5 using the miRCURY LNA Array miR labeling kit (Exiqon, Vedbaek, Denmark). Labeled RNAs were hybridized onto 3D-Gene Human miRNA Oligo chips (v.16.0; Toray Industries, Tokyo, Japan). The number of mounted miRNAs on this microarray is 1212 in total. Annotation and oligonucleotide sequences of the probes were conformed to the miRBase miRNA data base Release 16　(http://microrna.sanger.ac.uk/sequences/). After stringent washes, fluorescent signals were scanned with the 3D-Gene Scanner (Toray Industries, Japan) and analyzed using 3D-Gene Extraction software (Toray Industries, Japan). Microarray data is deposited as a MIAME compliant study in NCBI's Gene Expression Omnibus [30] and are accessible through GEO Series accession number GSE65033 (http://www.ncbi.nlm.nih.gov/geo/query/acc.cgi?acc=GSE65033).

### Normalized data processing and heatmap drawing.

The digitalized fluorescent signals provided by the software were regarded as the raw data. Individual miRNAs were regarded as present if the corresponding microarray signals were more than [the mean + 2x standard deviation] of the blank spot signals of which the top and bottom 5% ranked by signal intensity were removed. Once the miRNA was regarded as present, the miRNA signal was subtracted with the mean signal of the blank spots of which the top and bottom 5% ranked by signal intensity were removed. Next all the normalized data were globally normalized per microarray, such that the median of the signal intensity was adjusted to 25. miRNAs of which average of C group or that of FM group was more than 5 were selected for drawing the heatmap. The heatmap was drawn with R 3.0.2 * [31].

### microRNA RT-qPCR amplification

miRNA reverse transcription was performed with the miScriptII Reverse Transcription kit (Qiagen, cat. 218161) according to manufacturer´s instructions. cDNAs obtained were further amplified by quantitative PCR (qPCR) with the miScript SYBR Green PCR kit (Qiagen, cat. 218073) using the commercial product recommendations and a LightCycler 480 Real-Time PCR System (Roche, Switzerland) under standard amplification conditions, which included a single hotstart polymerase preactivation cycle at 94°C for 15 min, followed by 45 amplification cycles each one consisting of 3 steps: denaturation at 95°C for 15s, annealing at 50-60°C for 30s and extension at 70°C for 30s. Forward miRNA specific primers used are shown in Table 1. As a reverse primer the Universal Primer (UP) provided with the kit was used in all amplifications. U6 RNA amplification levels were used for the relative quantification of the miRNAs amplified.

**Table 1:** qPCR primers.

miRNA ID	3D-Gene v.16 Microarray ID	Accession*	miRNA mature sequence	Primer sequence	Tm (°C)
**hsa-miR-223-3p**	hsa-miR-223	MIMAT0000280	UGUCAGUUUGUCAAAUACCCCA	TGTCAGTTTGTCAAATACCC	50.3
**hsa-miR-451a**	hsa-miR-451	MIMAT0001631	AAACCGUUACCAUUACUGAGUU	AAACCGTTACCATTACTGAG	49.8
**hsa-miR-338-3p**	hsa-miR-338-3p	MIMAT0000763	UCCAGCAUCAGUGAUUUUGUUG	TCCAGCATCAGTGATTTTGT	52.1
**hsa-miR-143-3p**	hsa-miR-143	MIMAT0000437	UGAGAUGAAGCACUGUAGCUC	TGAGATGAAGCACTGTAGC	51.9
**hsa-miR-145-5p**	hsa-miR-145	MIMAT0000435	GUCCAGUUUUCCCAGGAAUCCCU	GTCCAGTTTTCCCAGGAATCC	55.7
**hsa-miR-21-5p**	hsa-miR-21	MIMAT0000076	UAGCUUAUCAGACUGAUGUUGA	TAGCTTATCAGACTGATGTT	47.9
**hsa-miR-1908-5p**	hsa-miR-1908	MIMAT0007881	CGGCGGGGACGGCGAUUGGUC	CGGCGGGGACGGCGATTGG	66.9
**hsa-miR-1260b**	hsa-miR-1260b	MIMAT0015041	AUCCCACCACUGCCACCAU	ATCCCACCACTGCCACC	57.9

*Accession in miRbase database http://www.mirbase.org/ [24,25]

### Statistical Analysis

Continuous data are expressed as means ± SD. Categorical variables are expressed in percentages and absolute values. Differences between the two groups were compared with the χ2 test or Fisher’s exact test for categorical variables, when necessary; for continuous data we used the Mann—Whitney U-test. To assess statistical dependence between quantitative variables we used the Spearman's rank correlation coefficient (rho). Differences between groups were considered significant if p < 0.05. Statistical analyses were performed with the SPSS package 13.0 (SPSS Inc, Chicago, IL, USA).

## Results

### Study population

Patients’ median age was 51 yrs. (range 39–63), all patients were female, had pain in both sides of their body as well as pain above and below their waist, with axial skeletal pain involvement. Median number of trigger points defined by the American College of Rheumatology (ACR) criteria [1] was 18 (range 14–18). Participants had suffered from FM for a median time of 18 yrs. (range 10–25). Total FIQ average score was 74.11±13.32 (range 45–95) and MFI´s for general fatigue was 18.64±1.63 (range 15–20). Physical fatigue scores were more prominent than mental fatigue’s with average scores of 17.64 ± 1.50 (range 15–19) and 14.27 ± 2.90 (range 8–19) respectively. For the reference group the only available data was median age: 47.5 yrs. (range 40–63) (S2 Table).

### Differences in miRNA expression profiles of FM patients and healthy participants

**Fig 1:**
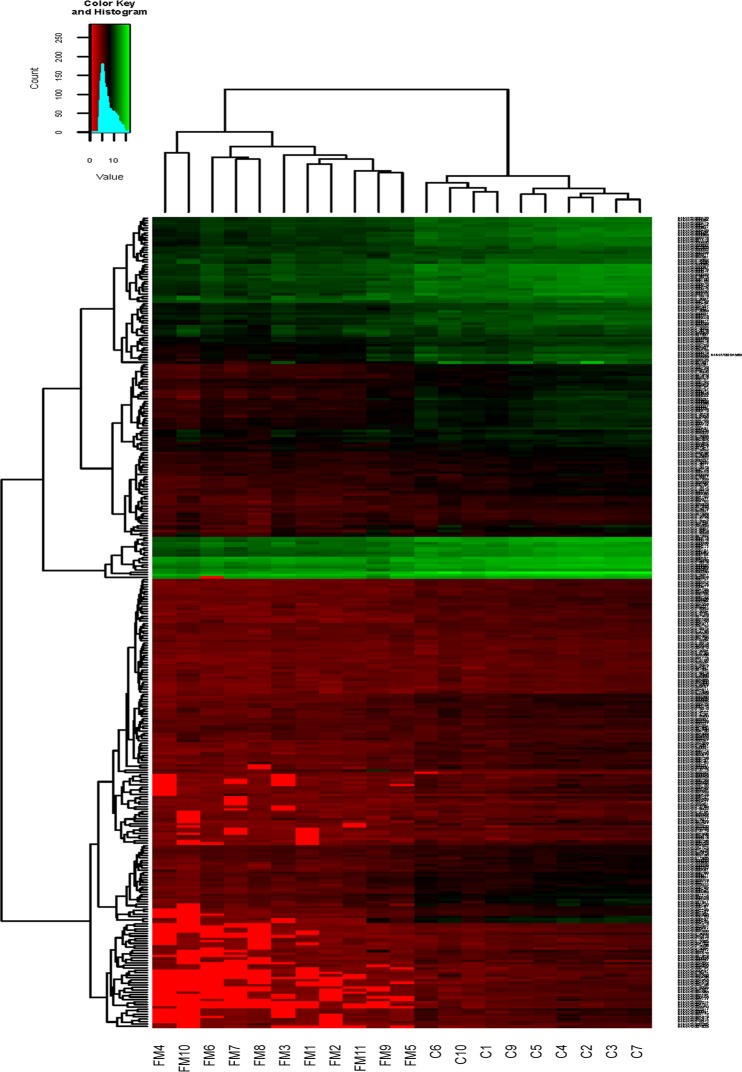
Heatmap of PBMCs miRNome from FM patients and controls. The microarray analysis was performed with 3D-Gene Human miRNA Oligo chips (v.16.0; Toray Industries, Tokyo, Japan), by using 3D-Gene Extraction software (Toray Industries, Japan) and the heatmap drawn with R 3.0.2 program [31]. Patient samples are labeled (FM 1–11) and controls (C 1–10). Color palette is included to indicate signal intensity.

Study of miRNA levels in PBMCs of 11 FM patients and 10 population age-paired healthy controls by microarray technology (3D-Gene Human miRNA Oligo chips v.16.0; Toray Industries, Tokyo, Japan), showed a marked reduction in the expression levels of a large subset of miRNA in patient PBMCs when compared to miRNA expression levels in control participant PBMCs. In particular 16% of the analyzed miRNAs (193/1212) showed inhibition of at least 50% in patients with respect to the level found in healthy controls and 3% of them (40/1212) a reduction of at least 75% when average values were compared. Another 3% (40/1212) were induced over 50% in patients, however, none of them reached over 3-fold induction. All but one of these upregulated miRNAs showed very low readings indicative of low abundance in PBMCs and the only upregulated miRNA with higher expression levels (hsa-miR3687) presented important inter-individual differences particularly in the FM group (Fig. 1 and microarray datasets GSE65033 (http://www.ncbi.nlm.nih.gov/geo/query/acc.cgi?acc=GSE65033)).

Of particular interest we found 5 miRNAs showing a 6 to over 13-fold inhibition in FM patients when compared to control values. These miRNAs corresponded to miRNAs hsa-miR-451a, hsa-miR-338-3p, hsa-miR-143-3p, hsa-miR-145-5p and hsa-miR-223-3p (Table 2).

**Table 2:** Microarray Average values after global normalization.

miRNA ID	Control Average	FM Average	Ratio C/FM Average
**hsa-miR-223-3p**	66736.98	10577.74	6.31
**hsa-miR-451a**	4830.07	358.62	13.47
**hsa-miR-338-3p**	374.20	32.22	11.62
**hsa-miR-143-3p**	506.26	45.42	11.15
**hsa-miR-145-5p**	546.05	52.58	10.39
**hsa-miR-21-5p**	5470.04	1409.50	3.88
**hsa-miR-1908-5p**	762.60	660.45	1.15
**hsa-miR-1260b**	23676.19	17356.79	1.36

### Small RNA levels were similar between fibromyalgia patients and controls

To discard the possibility of a sample group bias as an explanation to the general group differences observed in miRNA levels, the relative abundance of small RNA was determined in each of the analyzed samples, using small RNA kits (2100 Bioanalyzer, Agilent Technologies, USA). The results showed that on average the relative abundance of small RNA in each of the groups of participants under comparison were very similar: 14.0±3.4% in the control group and 16.1±10.2% in the patient group (S1 Table), and therefore could not account for the overall lower levels of expression of miRNAs found in patients and much less for the striking differences over 6-fold detected with the microarrays (Table 2).

### miRNA profile differences between FM patients and healthy participants by RT-qPCR amplification

In order to validate the findings of the microarray analysis, we evaluated expression of the most discriminating miRNAs, which corresponded to hsa-miR-451a, hsa-miR-338-3p, hsa-miR-143-3p, hsa-miR-145-5p and hsa-miR-223-3p by an alternative assay: retrotranscription followed by real-time PCR amplification or RT-qPCR. As control we selected other miRNAs showing practically same average values: hsa-miR-1908-5p and hsa-miR-1260b (Table 2). We also selected in our analysis a miRNA showing an intermediate control/FM ratio value of almost 4-fold (hsa-miR-21-5p) to evaluate if it could be confirmed by RT-qPCR.

**Fig 2:**
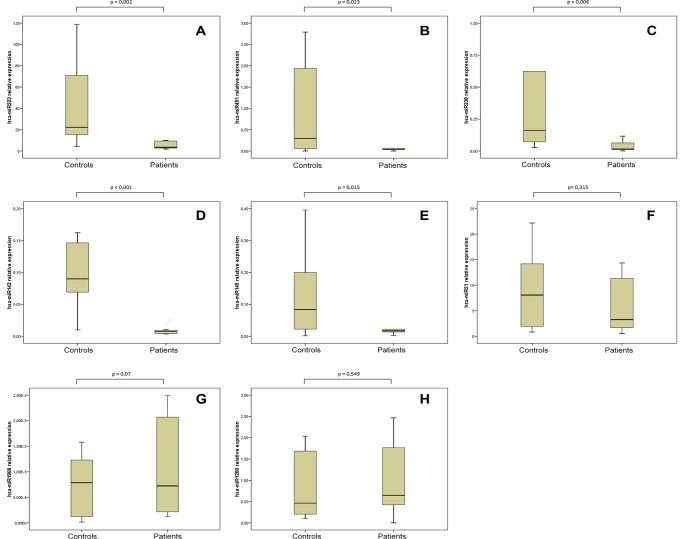
Boxplots showing qPCR validation of miRNome from FM patients and controls. Panels A-F show qPCR results for the miRNAs with striking downregulation (over 4-fold) in microarray analysis. Panels G and H show qPCR results for the miRNAs not downregulated in FM as per microarray analysis. P<0.05 was considered significant. Analysis was performed using the SPSS package 13.0 (SPSS Inc, Chicago, IL, USA).

As illustrated by box plotting, significant inhibition of miRNA levels between FM patients and controls could be appreciated for all miRNAs that had showed an average fold difference over 6 in microarray analysis. However, miRNAs with differences less than 4-fold did not appear significant by RT qPCR analysis, hsa-miR-21-5p included (Fig. 2 and S3 Table). Changes were considered significant when p value was <0.05.

### Correlation between miRNA expression levels and cardinal symptoms in FM patients

The relationship of each of the 5 significantly inhibited miRNAs in FM patient PBMCs with symptom severity was assessed by comparing miRNA levels with total FIQ and the scores of each of the five MFI subscales. All the p values obtained were >0.05 and therefore no correlation of miRNA levels in patients could be associated with the total FIQ scores or MFI subscales (Table 3).

**Table 3:** Correlation between miRNA expression levels and cardinal symptoms in FM patients.

Spearman	Parameters	miR-143-3p	miR-145-5p	miR-223-3p	miR-338-3p	miR-451a
	N	9	9	11	9	11
**FIQ**	rs	-0.19	0.14	0.03	0.10	-0.33
	p-value	0.69	0.74	0.75	0.73	0.71
**GF**	rs	0.03	0.08	0.13	0.005	0.09
	p-value	0.66	0.46	0.30	0.85	0.35
**FF**	rs	0.27	0.26	0.01	0.01	0.00
	p-value	0.15	0.16	0.76	0.85	0.90
**MF**	rs	-0.38	-0.37	0.01	-0.76	-0.58
	p-value	0.67	0.67	0.75	0.63	0.69
**RA**	rs	0.31	0.36	0.02	0.01	0.11
	p-value	0.12	0.08	0.70	0.80	0.31
**RM**	rs	0.32	0.37	0.15	0.28	0.00
	p-value	0.11	0.08	0.24	0.14	0.96

N (number of samples analyzed); r_s_ (Spearman rho coefficient); total FIQ: Fibromialgia Inventory Questionnaire (scale 0–100) covering three domains: function, overall impact, and symptoms [26,27]. GF: General Fatigue; PF: Physical and MF: Mental Fatigue, RA: reduced activity and RM: reduced motivation, are subscales of the MFI (Multidimensional Fatigue Inventory) (scale 0–20) [28]

However, it is interesting to note that patient FM9 who had the lowest mental fatigue score (8) compared to an average of 14.3±2.8 in the FM group (n = 11), presented the highest levels for 4 of the 5 markedly inhibited miRNAs: hsa-miR-143, hsa-miR145, hsa-miR223 and hsa-miR451, with values for the two first miRNAs above lowest levels in control subjects and with a less than two-fold difference for the other two miRNAs (hsa-miR223 and hsa-miR451) with respect to its corresponding reference value in the control group (lowest control level, S4 Table, (a) values), suggesting a possible correlation between some of these miRNAs and mental fatigue severity.

## Discussion

Even though the association of FM to particular viral infections has not been confirmed to date [8,9,14–17], the possibility of a virus being the etiologic agent or a contributor of disease progression in FM cannot be discarded. The study of PBMC FM miRNome should allow for a better understanding of FM patient immune system defects and their relation with recurrent viral infections. Viruses can manipulate the cellular processes necessary for their replication by targeting the host RNAi machinery [32–34]. Our work shows that differences in expression levels of a large subset of PBMC miRNA exist between FM patient and control groups. About 20% of the analyzed miRNAs showed at least 2-fold inhibition in the FM group while less than 0.5% showed upregulation to these levels (Fig. 1 and microarray datasets GSE65033 (http://www.ncbi.nlm.nih.gov/geo/query/acc.cgi?acc=GSE65033)) which may indicate miRNA synthetic pathway defects in FM patients, perhaps linked to viral infections. Interestingly, several reports link general downregulation of host miRNAs in response to human influenza A [35] or other viral [36–38] infections. Combinatorial contributions of multiple host miRNAs have also been described and shown to be strain and host-specific in addition to vary with time-post-infection [35–38]. For example, Sun *et al*. found that while microRNA-223 levels were significantly enriched in HIV-1-infected CD4(+)CD8(-) PBMCs, microRNA-29a/b, microRNA-155 and microRNA-21 levels were significantly reduced [36] and Terrier *et al*. that four miRNAs (miR-21, miR-29a, miR-29b and miR-452) were downregulated and only one (miR-146a) upregulated in lung A549 cells upon H1N1 and H3N2 infection [35]. Thus, chronic recurrent infections could potentially lead to defined miRNomes. However, at this point we do not know whether the combination of miRNAs found to be strikingly downregulated in FM patients is related to viral infections.

PBMC miRNA profile differences between the two groups could be due to differences in PBMC subpopulations, dependent or not on viral infections. Even though some groups of researchers have reported statistically significant differences in NK cells and some lymphocyte subtypes in FM patients [39–41], others report no differences in FM white blood cell counts [42,43]. Further analyses to clarify whether miRNA differences in FM patients are related to altered blood cell counts are granted.

Our analysis of FM patient miRNomes includes the complete collection of human miRNA sequences corresponding to the miRbase-version16 (1212 sequences), which almost doubles the number of sequences in one previous study (748 sequences) [22] and triplicates the number of miRNAs analyzed in a second study (374 sequences) [23]. In addition, this is to our knowledge, the first study in FM PBMCs and the only one in FM patients that includes validation of results by an alternative approach. Among the six miRNAs presenting a marked downregulation by microarray analysis, hsa-miR-21-5p inhibition could not be confirmed by qPCR and therefore is not proposed as a biomarker of FM. It is interesting to note that hsa-miR-21-5p was the one showing the mildest inhibition of the six selected from the microarray screen (Table 2).

Interestingly enough, two out of the five miRNAs we are proposing as biomarkers of FM, in particular hsa-miR223-3p and hsa-miRNA-145-5p, are inhibited in cerebrospinal fluid of FM patients [22]. PBMCs miRNome should not be expected to coincide with that of cerebrospinal fluid. In fact, those same authors reported the finding of a complete different battery of miRNAs showing differences in serum of FM patients with respect to control levels (seven showed downregulation: miR-103a, miR-107, let-7a-5p, miR-30b-5p, miR-151a-5p. miR-142-3p, miR-374b-5p while one showed up-regulation: miR-320a) [23]. In contrast, miRNome of plasmas from Chronic Fatigue Syndrome (CFS) patients, which share symptoms with FM patients, showed upregulation of miRNA-143-3p [44]. The fact that hsa-miR-223-3p and hsa-miRNA-145-5p have been associated to FM in more than one body fluid type strengthens our proposal of these miRNAs as biomarkers of the disease. It needs to be mentioned that hsa-miR-21 was found to be downregulated in the cerebrospinal fluid of FM patients by qPCR [22]. Further testing should allow clarifying this discrepancy.

In addition to viral infections, hsa-miR-21 and hsa-miR-223 have been linked to inflammation and injury of the central nervous system [45–48], hsa-miR-143-3p/hsa-miR-145-5p to muscle functioning [49,50] and hsa-miR338/hsa-miR-451 downregulation to diabetes and inflammation [51,52]. The significance of the five miRNAs we are proposing as FM biomarkers in relation to cardinal symptoms of the disease, however, can only be speculated at this point. Even though we aimed at finding correlations between miRNA patient levels and severity of symptoms our statistical analysis, contrary to the study in cerebrospinal fluid [22], did not detect any (Table 3). Our stringent selection criteria of patients leading to homogenous symptomatology in participants, together with the low number of samples analyzed may explain this result. In this sense, however, it was interesting to find that patient FM9 showing the lowest mental fatigue score (8) compared to the rest of the group (14,3±2,8) presented the highest levels for four of the five proposed FM biomarkers: hsa-miR-143-3p, hsa-miR145-5p, hsa-miR223-3p and hsa-miR451a; showing even higher levels than the lowest value of the control group for the two first miRNAs and a difference of less than 2-fold in the other two miRNAs, with respect to the corresponding reference value (S4 Table, (a) values). This suggests a possible correlation between these four miRNAs and severity of mental fatigue. Being this the case, analysis of the proposed FM biomarker miRNAs in larger cohorts of patients with differences in cardinal symptoms should lead to identification of correlations between particular miRNAs and specific FM symptoms or symptom severity.

Depression is one of the well-known symptoms of FM patients and circulating miRNAs are now being considered as possible biomarkers in depression pathogenesis and in monitoring therapeutic responses [53]. However, some of the miRNAs found to be upregulated in PBMCs of patients with major depressive disorder (MDD), such as hsa-miRNA-26b and hsa-miRNA-1972 [54] are not upregulated in our cohort of FM patients (GEO datasets GSE65033 (http://www.ncbi.nlm.nih.gov/geo/query/acc.cgi?acc=GSE65033)). Interestingly enough, the studies recently performed by O´Connor *et al*., in a rat model of depression induced by early-life stress, show a marked inhibition of the miRNA-451 that could be attenuated by antidepressant treatment [55] suggesting that some of our FM patient symptoms may improve with this type of medication possibly through mechanisms involving miRNA-451.

## Conclusions

Downregulation of a large subset of miRNAs (about 20% of the 1212 sequences analyzed) observed in FM patients could be indicative of recurrent viral infections. However we do not count at present with evidence to support this hypothesis.

Although this general feature could be a distinctive trait of FM patients we propose the use of the five validated miRNAs, which include hsa-miR223-3p, hsa-miR451a, hsa-miR338-3p, hsa-miR143-3p and hsa-miR145-5p for the diagnosis of the disease since the drastic differences detected in patients (at least 6-fold inhibited) should allow for the development of a more sensitive assay. Population validation of these biomarkers in larger study groups are at need.

## Supporting Information

S1 Table Concentration, integrity and purity of analyzed RNA samples. (PDF)

S2 Table Demographic data of participants in the study. (PDF)

S3 Table RT qPCR C_t_ values analysis. (PDF)

S4 Table Microarray readings after global normalization. (PDF)
