# Supplementary material for: Identification of a MicroRNA Signature for the Diagnosis of Fibromyalgia
Source: PLoS One. 2015 Mar 24;10(3):e0121903. doi: 10.1371/journal.pone.0121903 (PMC4372601; doi:10.1371/journal.pone.0121903)
Supplement: S1 Table — (PDF) [file pone.0121903.s001.pdf]

Table S1. Concentration, integrity and purity of analyzed RNA samples

Sample	Concentration ng/µl	A_{260}/A_{280}	A_{260}/A_{230}	RIN	miRNA (%)
**C1**	168.0	1.98	2.31	ND	10
**C2**	182.5	2.03	1.62	8.1	12
**C3**	187.4	2.06	2.17	8.4	15
**C4**	341.9	2.06	2.05	8.3	15
**C5**	195.4	2.02	2.15	7.4	15
**C6**	184.8	2.00	2.20	8.3	13
**C7**	187.1	2.07	2.14	8.5	15
**C8**	200.1	1.94	2.15	ND	21
**C9**	189.0	2.02	2.24	7.2	16
**C10**	191.6	2.02	2.2	7.7	8
**FM1**	176.6	1.94	2.15	ND	14
**FM2**	183.6	1.94	2.14	10	16
**FM3**	185.3	1.94	1.86	10	11
**FM4**	166.9	1.93	2.15	10	39
**FM5**	188.0	1.96	1.49	ND	18
**FM6**	165.5	1.94	2.19	10	4
**FM7**	118.8	1.92	2.27	ND	16
**FM8**	164.7	1.96	2.15	10	19
**FM9**	179.2	1.93	2.10	ND	30
**FM10**	177.0	1.91	2.22	ND	6
**FM11**	174.0	1.93	2.13	ND	4

C indicates control samples (healthy individuals) and FM patient samples.
ND indicates not determined.
