# Supplementary material for: Identification of a MicroRNA Signature for the Diagnosis of Fibromyalgia
Source: PLoS One. 2015 Mar 24;10(3):e0121903. doi: 10.1371/journal.pone.0121903 (PMC4372601; doi:10.1371/journal.pone.0121903)
Supplement: S2 Table — (PDF) [file pone.0121903.s002.pdf]

**Table S2.** Demographic data of participants in the study

| Patient | Age | Sex | TP | FIQ | GF | PF | MF | RA | RM | Control | Age |
|---|---|---|---|---|---|---|---|---|---|---|---|
| **FM1** | 47 | F | 16 | 76.50 | 19 | 19 | 16 | 14 | 16 | C1 | 46 |
| **FM2** | 51 | F | 18 | 84.19 | 18 | 19 | 14 | 15 | 14 | C2 | 61 |
| **FM3** | 55 | F | 18 | 74.96 | 20 | 18 | 13 | 10 | 14 | C3 | 47 |
| **FM4** | 52 | F | 18 | 94.19 | 20 | 19 | 13 | 20 | 19 | C4 | 40 |
| **FM5** | 63 | F | 18 | 82.57 | 20 | 19 | 14 | 19 | 13 | C5 | 42 |
| **FM6** | 57 | F | 18 | 68.23 | 19 | 17 | 12 | 16 | 16 | C6 | 48 |
| **FM7** | 40 | F | 18 | 80.62 | 20 | 17 | 16 | 15 | 17 | C7 | 50 |
| **FM8** | 40 | F | 18 | 78.12 | 15 | 19 | 17 | 15 | 10 | C8 | 63 |
| **FM9** | 48 | F | 18 | 70.94 | 17 | 16 | 8 | 9 | 9 | C9 | 47 |
| **FM10** | 55 | F | 18 | 45.14 | 18 | 15 | 12 | 5 | 8 | C10 | 59 |
| **FM11** | 39 | F | 18 | 57.65 | 18 | 17 | 15 | 14 | 12 | - | - |

F: Female; TP: Tender Points; total FIQ: Fibromialgia Inventory Questionnaire (scale 0-100) covering three domains: function, overall impact, and symptoms [26,27]. GF: General Fatigue; PF: Physical and MF: Mental Fatigue, RA: reduced activity and RM: reduced motivation, are subscales of the MFI (Multidimensional Fatigue Inventory) (scale 0-20) [28]
