# Supplementary material for: Identification of a MicroRNA Signature for the Diagnosis of Fibromyalgia
Source: PLoS One. 2015 Mar 24;10(3):e0121903. doi: 10.1371/journal.pone.0121903 (PMC4372601; doi:10.1371/journal.pone.0121903)
Supplement: S3 Table — (PDF) [file pone.0121903.s003.pdf]

Table S3. RT qPCR C_t values analysis

miRNA	FM Median (-ΔCt)	C Median (-ΔCt)	ΔΔCt	Fold change ($2^{ΔΔCt}$)	p value
**hsa-miR-223-3p**	22,1	17.8	4.3	19.7	0.002
**hsa-miR-451a**	30.0	23.8	6,2	73.5	0.023
**hsa-miR-338-3p**	30.7	24.1	6.6	97.0	0.008
**hsa-miR-143-3p**	31.5	25.4	6.1	68.5	0.000
**hsa-miR-145-5p**	31.0	25.4	5.6	48.5	0.015
**hsa-miR-21-5p**	23.1	20.1	3.0	8.0	0.315
**hsa-miR-1908-5p**	25.8	22.9	2.9	7.46	0.070
**hsa-miR-1260b**	36.4	32.9	3.5	11.3	0.549

$ΔC_t$ increment in cycle threshold
