# Supplementary material for: Identification of a MicroRNA Signature for the Diagnosis of Fibromyalgia
Source: PLoS One. 2015 Mar 24;10(3):e0121903. doi: 10.1371/journal.pone.0121903 (PMC4372601; doi:10.1371/journal.pone.0121903)
Supplement: S4 Table — (PDF) [file pone.0121903.s004.pdf]

**Table S4.** Microarray readings after global normalization

| Sample | hsa-miR-451a | hsa-miR-338-3p | hsa-miR-143-3p | hsa-miR-145-5p | hsa-miR-223-3p |
|---|---|---|---|---|---|
| **C1** | 2227.49 | 264.16 | 375.81 | 473.62 | 51653.59 |
| **C2** | 19593.32 | 267.72 | 582.35 | 567.22 | 61996.62 |
| **C3** | 3301.84 | 550.42 | 781.25 | 852.59 | 77138.78 |
| **C4** | 3730.19 | 428.65 | 815.75 | 824.65 | 72748.41 |
| **C5** | 1146.57 | 343.73 | 492.04 | 589.58 | 77202.88 |
| **C6** | 683.43 [(a)] | 199.51 | 319.85 | 380.56 | 39896.83 |
| **C7** | 1889.09 | 743.85 | 604.23 | 638.50 | 88579.29 |
| **C8** | 7335.51 | 357.20 | 502.17 | 484.69 | 86658.50 |
| **C9** | 5135.83 | 459.92 | 474.34 | 533.64 | 76955.84 |
| **C10** | 3257.46 | 126.84 [(a)] | 114.76[(a)] | 115.43 [(a)] | 34539.07 [(a)] |
| **FM1** | 305.83 | 26.32 | 15.18 | 18.56 | 10111.74 |
| **FM2** | 206.56 | 32.83 | 16.00 | 16.11 | 11116.45 |
| **FM3** | 1734.45 | 32.14 | [(b)] | [(b)] | 7050.56 |
| **FM4** | 161.66 | [(b)] | 20.37 | 28.65 | 6314.36 |
| **FM5** | 330.34 | 53.04 | 82.15 | 75.43 | 16817.25 |
| **FM6** | 202.13 | 16.34 | 24.65 | 25.40 | 9053.61 |
| **FM7** | 78.32 | 17.96 | 22.41 | 20.47 | 5860.54 |
| **FM8** | 119.19 | 19.82 | 16.27 | 38.76 | 11742.76 |
| **FM9** | 337.02 | 47.76 | 194.84 | 233.92 | 19213.68 |
| **FM10** | 259.32 | [(b)] | [(b)] | [(b)] | 4221.95 |
| **FM11** | 210.06 | 43.73 | 16.92 | 15.92 | 14852.25 |

(a) lowest values in control individuals

(b) no readings above background obtained
